# The effects of digoxin on heart failure mortality and re-admission in a single center cross-sectional study

**DOI:** 10.34172/jcvtr.33062

**Published:** 2024-09-20

**Authors:** Mahsa Behnemoon, Zahra Borumandkia

**Affiliations:** ^1^Department of Cardiology, School of Medicine, Urmia University of Medical Sciences, Urmia, Iran; ^2^Medical Center, Urmia University of Medical Sciences, Urmia, Iran

**Keywords:** Heart failure, Digoxin, Mortality, Readmission

## Abstract

**Introduction:**

Mortality benefit of digoxin prescription in patients suffering from heart failure has been questioned many time. We evaluated these effects among admitted symptomatic heart failure patients.

**Methods:**

We retrospectively divided our patients into two groups: group A (n=205) were digoxin prescribed, and group B (n=96) were digoxin naïve patients. Both groups’ medical records were gathered for one year, and the study endpoints were compared between the two groups.

**Results:**

The mean age was 62.3±12.1 years and 54.8 % were male. All-cause mortality and readmission occurred in 26.7% and 31.7% of individuals, respectively, without significant differences between the two groups. However, in subgroup analysis, there was a significant relationship between in-hospital mortality and the presence of cardiovascular risk factors.

**Conclusion:**

Digoxin might increase in-hospital mortality in patients with underlying cardiovascular risk factors.

## Introduction

 Heart failure is the leading cause of hospitalization in individuals over 65 years of age, and is a costly health problem with high rates of mortality and morbidity.^1-3^ Digoxin is considered to have neutral or even beneficial effects on mortality according to some large studies.^4,5^ Nevertheless, some trials have mentioned its adverse effects on all-cause mortality with no significant efficacy on heart failure readmission rate.^6-11^ Besides the effects of digoxin may be related to its serum concentration according to some studies.^7^ However, the drug serum level is not accessible in some regions and some patients ignore serial laboratory monitoring. In this brief study we evaluated the effects of digoxin on short-term, one-year mortality, and re-admission rate among patients admitted with symptomatic heart failure and reduced ejection fraction (HFrEF) during 2018-2019.

## Materials and Methods

 In this single-center cross-sectional study, all HFrEF patients who were admitted to our cardiology department during 2018-2019 were enrolled in the study. Patients should fulfill all these criteria: age above 18 years, ejection fraction below 40%, class III-IV symptoms (NYHA III-IV), sinus rhythm or rapid response atrial fibrillation (AF), access to the admission data, and one-year follow-up medical records. 600 patients enrolled in the study, of which 356 cases fulfilled our inclusion criteria and were retrospectively divided into two separate groups: in group A (n = 205) patients, digoxin was prescribed during hospitalization for atrial fibrillation or heart failure symptoms, and its administration was continued after discharge. Group B were digoxin naive patients during hospitalization and after discharge (n = 151). Sex, age, underlying disease (diabetes, hypertension, ischemic heart disease, renal failure), and heart failure medications were not different significantly between the two groups. Both groups’ medical records were gathered for one year, and in case of missing data, telephone contact was made with the patients or their family members. Finally, in-hospital mortality, readmission rate, and all-cause mortality during one-year follow-up were compared between the two groups. All analyses were performed using IBM SPSS software for windows version 22, and the p-value of < 0.05 was considered significant.

## Results

 The mean age of participants was 62.3 ± 12.1 years, and 54.8 % were male. Heart failure had ischemic etiology in 28.3% of cases, and hypertension was the most common comorbidity (53.9%). Atrial fibrillation was seen in approximately 20% of cases, and most individuals were admitted due to decompensated heart failure. De novo HF was present in only 9% of cases. Patients with coronary artery disease, hypertension, and diabetes mellitus had significantly lower ejection fraction compared to those without comorbidities or other underlying diseases (*P* = 0.005). (Table 1)

**Table 1:** Baseline characteristics of study population.

**Variables**		**Number (%)**	**Group A**	**Group B**	***P*** ** value**
Sex	male	195 (54.8)	115	80	0.32
	female	161 (45.2)	90	71	
Age	< 65 65 <	115 (32.3) 241 (67.7)	63 142	52 99	0.342
Underling disease	Hypertension	192 (53.9)	103	89	0.091
	Coronary artery diseases	101 (28.3)	56	45	
	Diabetes mellitus	92 (25.8)	49	43	
	Chronic renal disease	26 (7.3)	9	17	
	Obstructive pulmonary disease	24 (6.7)	13	11	
	Cerebrovascular disease	11 (3.1)	4	7	
	Thromboembolic disease	8 (2.2)	5	3	
	No underlying disease	80 (22.4)	51	29	
Smoking	Yes	124 (34.8)			
	No	232 (65.2)			
Ejection fraction	20% >	117 (32.8)	80	37	0.076
	20-40%	239 (67.1)	125	114	
Reason for hospitalization	ADHF + sinus rhythm	283 (79.5)	159	124	0.23
	ADHF + Rapid response AF	73 (20.5)	46	27	

ADHF: acute decompensated heart failure, AF: atrial fibrillation

 During hospitalization, we observed a 3.4% mortality rate. Major adverse cardiac events occurred among 62.1% (n = 209) of discharged subjects during the follow-up period. All-cause mortality and readmission rate were 26.7% (n = 96) and 31.7% (n = 113), respectively, without significant difference between the two study groups. There was no significant correlation between digoxin administration and study endpoints. However, in a subgroup analysis, we noticed increased in-hospital mortality among patients suffering from hypertension, diabetes mellitus, or coronary artery disease who had been prescribed digoxin during hospitalization (*P* = 0.048). (Table 2)

**Table 2:** Treatment outcomes according to the age, sex, underlying disease, ejection fraction and the cause of hospitalization

		**In-hospital mortality (n=12)**			**One-year mortality (n=96)**			**Readmission (n=113)**		
		**Group A** **(n=9)**	**Group B** **(n=3)**	***P *** **value** **(0.21)**	**Group A** **(n=52)**	**Group B** **(n=44)**	***P*** ** value** **(0.42)**	**Group A** **(n=66)**	**Group B** **(n=47)**	***P*** ** value** **(0.83)**
Age < 65 > 65		2 (3.2%)	2 (3.8%)	0.84	8 (12.7%)	8 (15.4%)	0.67	29 (46%)	20 (38.5%)	0.41
		7 (4.9%)	1 (1%)		44 (31%)	36 (36.4%)		37 (26.1%)	27 (27.3%)	
Sex Male Female		6 (5.2%)	3(3.75%)	0.67	31 (26.5%)	20 (25.6%)	0.89	36 (30.8%)	25 (32.1%)	0.85
		2 (2.2%)	1 (1.4%)		22 (25%)	23 (26.1%)		30 (34.1%)	22 (30.6%)	
At least one cardiovascular risk factor (DM, HTN, IHD)	yes	7 (5.1%)	1 (0.8%)	0.048	37 (27.2%)	36 (30%)	0.65	49 (36%)	39 (32.5%)	0.90
	no	2 (3.9%)	2 (6.8%)		15 (21.7%)	8(25.8%)		17 (24.6%)	8 (25.8%)	
EF < 10% 10-20% 20-30% 30-40%		0	0	N/A	0	1 (33.3%)	N/A	0	1	N/A
		4 (5.2%)	1 (2.8%)	0.56	23 (29.9%)	11 (28.3%)	0.82	20 (26%)	13 (36.1%)	0.27
		2 (2.9%)	1 (1.6%)	0.65	16 (22.9%)	22 (36.7%)	0.08	28 (40%)	15 (25%)	0.07
		3 (5.3%)	1 (1.9%)	0.35	12 (21.1%)	11 (21.2%)	0.99	18 (36.1%)	18 (34.6%)	0.73
Cause of hospitalization	ADHF + NSR (N = 283)	7 (4.4%)	3 (1.98%)	0.24	36 (24.5%)	38 (27.9%)	0.5	48 (32.7%)	42 (30.8%)	0.85
	AF(RVR) (N = 73)	2 (4.3%)	0	0.58	16 (36.3%)	6 (54.6%)	0.61	18 (41%)	5 (45.5%)	0.37

DM: diabetes mellitus, HTN: hypertension, IHD: ischemic heart disease, EF: ejection fraction, ADHF: acute decompensated heart failure, AF: atrial fibrillation, RVR: rapid ventricular response

## Discussion

 We evaluated the possible effects of digoxin on in-hospital and one-year mortality and re-admission rates of HFrEF patients. Based on the latest consensus recommendations, digoxin administration can be beneficial for HFrEF patients who are symptomatic despite optimal doses of standard HF treatment.^4,5^ Nevertheless, the Valsartan Heart Failure Trial has shown that consumption of digoxin is related to higher mortality risk and readmission due to worsening heart failure.^9^ In addition, a retrospective study on advanced heart failure patients showed increased risk of mortality, emergency heart transplantation, or need for a ventricular assist device in patients treated with digoxin.^10^ According to the post hoc analysis of the DIG trial, digoxin serum level and mortality rate were higher among female patients as compared to men.^11^ However, our study failed to show any association between sex and the effect of digoxin on mortality due to different in terms of methodology, sample size and baseline patients’ characteristics. The fact that digoxin can have different effects on clinical improvement or readmission rate among male and female patients, is not fully understood.

 In our study we didn’t notice any significant relationship between taking digoxin and mortality or readmission, in line with Dhaliwal et al’s study.^12^ According to the study of Ahmed and colleagues, digoxin reduced the one-month readmission rate and its beneficial effects lasted for the rest of 12 months during follow-up.^13^ Another study done by Gheorghiade et al showed that digoxin can improve outcomes in the subgroup of NYHA class III–IV and left ventricular ejection fraction (LVEF) below 25%.^14^ However, we didn’t find a significant difference in the outcome according to their LVEF and interestingly, we noticed increased risk of in-hospital mortality in a subgroup of patients with underlying cardiovascular risk factors. Electrolyte imbalance commonly seen in these subgroups of patients, in addition to the poly-pharmacy and higher risk of drug interactions, could explain our results. We suggest that in cases of limited access or cost issues of monitoring digoxin serum level, selective laboratory monitoring during hospital stay in these high-risk subgroups may be beneficial and cost-effective.

## Conclusion

 Digoxin had neutral effect on readmission and mortality of HFrEF patients, and its prescription might increase in-hospital mortality in patients with underlying cardiovascular risk factors.

## Competing Interests

 None.

## Ethical Approval

 This research is conducted in accordance with the principles set forth in the Helsinki Declaration 2008 and is accepted in the Ethics Committee of Urmia University of Medical Science in December 2018 which waived the informed consent due to its observational nature. The approval ID is IR.UMSU.REC.1396.519.
